# Radial Profile Analysis of Epithelial Polarity in Breast Acini: A Tool for Primary (Breast) Cancer Prevention

**DOI:** 10.3389/fmed.2019.00314

**Published:** 2020-01-10

**Authors:** Lawton Manning, Julia Holmes, Keith Bonin, Pierre-Alexandre Vidi

**Affiliations:** ^1^Department of Physics, Wake Forest University, Winston-Salem, NC, United States; ^2^Department of Cancer Biology, Wake Forest School of Medicine, Winston-Salem, NC, United States; ^3^Comprehensive Cancer Center of Wake Forest University, Winston-Salem, NC, United States

**Keywords:** breast cancer, organoids, apical polarity, chemoprevention screening, toxicology

## Abstract

Preventing cancer is vastly better than treating the disease in terms of a patient's quality of life and healthcare costs. Yet, to screen for chemopreventative drugs or evaluate interventions aimed at lowering cancer risk, quantitative readouts of risk are needed. In the breast and in other organs of epithelial origin, apical-basal polarity is key to homeostasis and is one of the first tissue characteristics lost during cancer initiation. Therefore, apical-basal polarity may be leveraged as an “architectural” determinant of cancer risk. A classic approach to quantify the localization of epithelial polarity markers is visual scoring at the microscope by trained investigators. This approach is time-intensive and limited to low throughput. To increase the speed, accuracy, and scoring volume, we developed an algorithm that essentially replaces the human eye to objectively quantify epithelial polarity in microscopy images of breast glandular units (acini). Acini in culture are identified based on a nuclear stain and the corresponding masks are divided into concentric terraces of equal width. This positional information is used to calculate radial intensity profiles (RP) of polarity markers. Profiles with a steep slope represent polarized structures, whereas more horizontal curves are indicative of non-polarized acini. To compare treatment effects, RP curves are integrated into summary values of polarity. We envision applications of this method for primary cancer prevention research with acini organoids, specifically (1) to screen for chemoprevention drugs, (2) for toxicological assessment of suspected carcinogens and pharmacological hit compounds, and (3) for personalized evaluation of cancer risk and risk-reducing interventions. The RadialProfiler algorithm developed for the MATLAB computing environment and for users without prior informatics knowledge is publicly available on the Open Science Framework (OSF).

## Introduction

### Epithelial Polarity in the Normal Mammary Gland

**Figure 1:**
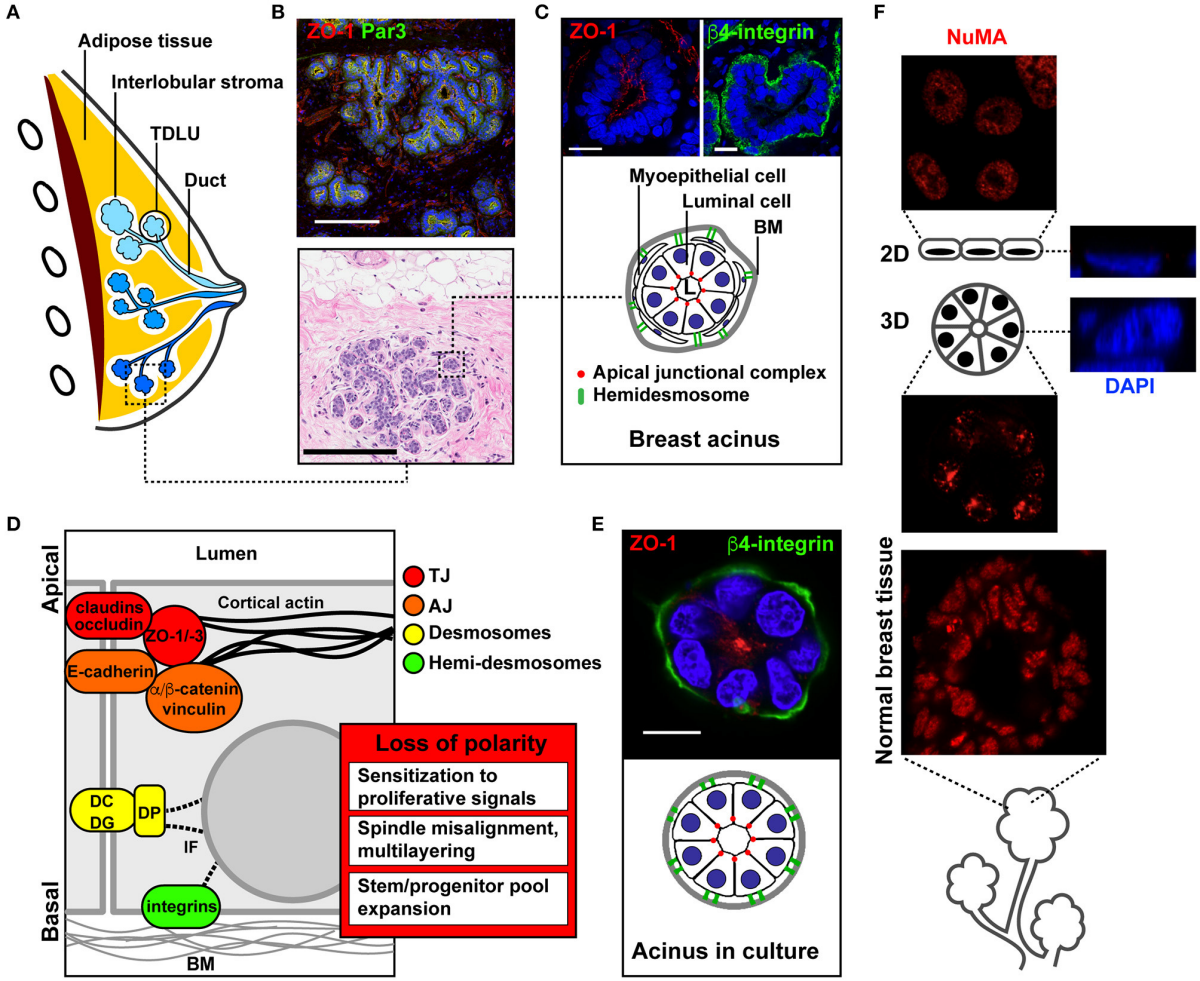
Apical-basal polarity in the normal mammary gland and in culture models of acini. **(A)** Schematic of the breast anatomy. Different ductal systems (or lobes) are shown in distinct shades of blue. TDLU, terminal ductal lobular unit (the initiation site for most breast carcinomas). **(B)** Immunohistochemistry (IHC, bottom) and immunofluorescence (IF, top) images of normal breast tissue sections. **(C)** Schematic and higher magnification images of functional glandular units (acini) stained with the ZO-1 and β4-integrin epithelial polarity markers. **(D)** Schematic of cell junctional complexes along the apical-basal polarity axis of the epithelium, and functional effects of epithelial polarity loss in cancer initiation. BM, basement membrane; DC, desmocollin; DG, desmoglein; DP, desmoplakin; IF, intermediate filaments. **(E)** Schematic and representative confocal images of a breast acinus produced in 3D culture. The IHC image in B is from the Komen Tissue Bank. Scale bars, 200 μm **(B)** and 20 μm **(C,E)**. **(F)** Immunostaining for the structural nuclear protein NuMA in a 2D monolayer culture (top), in a 3D culture of acini (middle), and in normal human breast tissue (bottom). Orthogonal views of stained nuclei (DAPI) in 2D and 3D cultures are shown on the right.

The mammary gland consists of an arborescence of ducts connecting the glandular elements (called acini, lobules, or alveoli) to the nipple (Figures 1A,B). Several (five to ten) of these ductal systems are typically present in each breast (1). The mammary gland is a simple epithelial tissue composed of a single layer of luminal cells lining the ducts and acini (Figure 1C). Luminal cells are surrounded by myoepithelial cells with contractile function to expel the milk toward the nipple. Myoepithelial cells also secrete most of the factors constituting the basement membrane (BM), a specialized form of extracellular matrix (ECM) lining the epithelium and rich in collagen type IV and laminins. In mammary ducts and acini, apical-basal polarity structurally and functionally defines the cellular organization relative to the lumen and BM (2, 3). Apical membranes of luminal cells delineate the luminal space and are segregated from basolateral membranes by cell-cell junctions; these different junctional complexes occupy distinct radial positions along the apical-basal polarity axis of the epithelial layer (Figure 1D).

Tight junctions (TJs) are localized closest to the lumen. They consist of integral membrane proteins [claudins, occludin, JAM (4)], as well as cytosolic adaptor and scaffolding factors [zona occludens proteins ZO-1, ZO-2, ZO-3 (5)] bridging the membrane-integral TJ factors with the cytoskeleton. TJs form a seal ensuring the segregation of apical and basolateral membrane lipids and proteins. In addition to this fence function, TJs serve as gates for selective diffusion between basal and luminal interstitial spaces. Both gate and fence functions are essential for the normal function of the gland, in particular for milk secretion and to control paracellular exchanges between blood and milk (6).

Adherens junctions (AJs) are located next to TJs and are composed of transmembrane cadherins and nectins bound to cytosolic catenins and to afadin. AJs provide attachment of neighboring cells and are physically bound to TJs via ZO-1. During cell differentiation, AJ formation precedes and promotes TJ assembly by nucleating TJ proteins (7, 8). Both TJs and AJs are connected to the actin cytoskeleton, with ZO proteins and catenins directly binding to and organizing F-actin, which leads to the establishment and maintenance of perijunctional actomyosin rings stabilizing junctional complexes (9, 10).

Desmosomes have a similar organization as AJs but, in contrast to AJs that are linked to actin filaments, desmosomes are connected to keratin intermediate filaments. Desmosomes also play an important role in cell-cell adhesion along the basolateral membrane. Together with AJs, desmosomes mechanically couple neighboring epithelial cells, and thereby provide mechanical strength to the tissue, define cell-intrinsic mechanical properties, and constitute mechanotransduction hubs for the integration of physical cues from surrounding cells (11, 12).

Cell-cell contacts in the breast epithelium and other epithelia also comprise gap junctions (GJs) that form channels connecting the cytoplasm of adjacent cells and that enable cell-cell communication via small molecules (13). GJs consist of connexons (connexin hexamers) and are classically represented toward the basal side of epithelial cells. Yet connexin 43 was recently found to be apically localized in the breast epithelium, and to be required for apical polarity establishment and maintenance (14).

Three major polarity complexes regulate the maturation and maintenance of cell-cell adhesion complexes along the apical-basal axis [reviewed in (2, 7)]: the crumbs complex, which defines apical membrane identity, the PAR (partitioning defective) system, which defines the apical-basal boundary, and the scribble complex, which defines basolateral membrane identity. The establishment of the apical-basal polarity axis—and particularly, the orientation of this axis orthogonal to the BM—also depends on cell-ECM interactions, which are critical for differentiation and homeostasis (15, 16). Such cell-ECM contacts involve both luminal and myoepithelial cells and are largely mediated by integrins located at the basal pole of the acini and ducts. Integrins cross-talk with and modulate growth factor receptors signaling, and play important roles in mechanosensing (17–19). Importantly, these ECM receptors initiate a structural continuum between the ECM and the cell nucleus, which defines nuclear shape and genomic functions (20).

As alluded to in the previous paragraphs, the function and relevance of cell-cell junctional complexes and cell-ECM contacts go far beyond their structural role. Polarity factors include tumor suppressors and oncoproteins that localize both at cell-cell junctions and in the cytosol or cell nucleus where they modulate biochemical signals, gene expression, and genome maintenance (21–23). Altered cell polarity causes misregulation of proliferative and survival pathways by shifting the proportion of soluble and membrane bound polarity factors. We also found evidence that cell-ECM interactions are required for an efficient DNA damage response in breast epithelial cells (24). Apical-basal polarity, specifically the PAR system, also defines the orientation of mitotic spindle poles, and hence the relative position of the daughter cells after cytokinesis; spindle orientation parallel to the BM is necessary for the maintenance of a single cell layer and, accordingly, epithelial polarity loss may promote cell multilayering and hyperplasia (25, 26). Epithelial polarity may therefore be considered an architectural biomarker of breast cancer risk and, indeed, disruption of epithelial polarity is one of the first identifiable events and a necessary step for the initiation of carcinoma (7, 27–29).

### Epithelial Polarity for Breast Cancer Risk Assessment

Current breast cancer risk assessment methods, such as the Gail model (30) provide population-based estimates of risk. Several genetic breast cancer risk factors have been identified (BRCA1, BRCA2, p53, etc.), yet the majority of breast cancers still have no clear germline mutation origin and cannot be predicted by genetic testing. Molecular assays of breast cancer risk are therefore needed for primary breast cancer prevention research and, ultimately, for personalized cancer prevention.

We propose that breast epithelial polarity, which is a hallmark of homeostasis in the mammary gland, is one of the molecular links between metabolic risk factors (including obesity and pre-diabetes) and cancer initiation. As such, epithelial polarity readouts may provide valid estimates of cancer risk. Loss of epithelial polarity, and in particular TJ and AJ remodeling, is associated with cancer initiation in multiple contexts, often involving tissue inflammation. For example, ulcerative colitis and Crohn's disease are both associated with elevated colorectal cancer risk (31) and are characterized by TJ dysfunctions (32). Similarly, patients with Celiac disease have TJ defects and increased epithelial cell permeability in the small intestine. These patients are at increased risk for adenocarcinoma of the small intestine. For breast cancer, obesity is one of the few modifiable risk factors and is characterized by a chronic state of inflammation and deregulation of cytokine and growth factors in circulation (33, 34). Our group found that cell microenvironments characteristic of obesity lead to the mislocalization of apical polarity proteins and premalignant changes in the mammary gland (14, 35). Apical polarity was also found to be disrupted by omega-6 fatty acids, which may be associated with increased breast cancer risk (36). These observations validate the concept of using epithelial polarity as a readout for primary prevention.

### Cell Culture Models of Breast Acini

When cultured with a reconstituted basement membrane (rBM) hydrogel having physical and chemical characteristics similar to that of the basement membrane *in vivo*, non-neoplastic mammary epithelial cells develop into 3D structures resembling mammary gland acini (Figure 1E). Acini cultures recapitulate important characteristics of the normal mammary gland, namely single cell-layered structures, proliferation arrest (90–95% Ki67-negative cells) and apical-basal polarity (37, 38). Signaling pathways are dramatically rewired in 3D acini cultures (39). Moreover, nuclear organization features, such as gene positioning and nucleoskeletal arrangement are strikingly different in acini cultures compared to 2D monolayer cultures (40, 41). Figure 1F illustrates a remarkable parallel between distribution patterns of a structural nuclear protein (NuMA) in normal breast tissue and acini cultures.

Mammary epithelial cells can be cultured either embedded in or on top of rBM (38). Micropatterned surfaces have also been developed as an alternative for acinar cultures (42). Acini cultures have the advantage of high reproducibility and manipulability. Compared to mouse models, experiments with acini cultures are cheaper, faster, raise fewer ethical concerns, and typically do not require regulatory approval. A limitation of classic breast acini cultures is the lack of other cell types (myoepithelial cells, fibroblasts, immune cells, adipocytes). Hence, experimentation with acini cultures does not replace, but complements, *in vivo* studies.

In principle, acini models can be used for high-content analyses (HCA) at medium to high throughput—“high-content” referring to complex phenotypic readouts. While many screening platforms have been developed around cancer models to identify new cancer treatments, HCA protocols with normal cells for cancer prevention are scarce. Obviously, readouts based on cell killing cannot be used in the context of prevention. HCA methods to assess epithelial polarity will contribute to fill this gap.

## The RadialProfiler Algorithm

**Figure 2:**
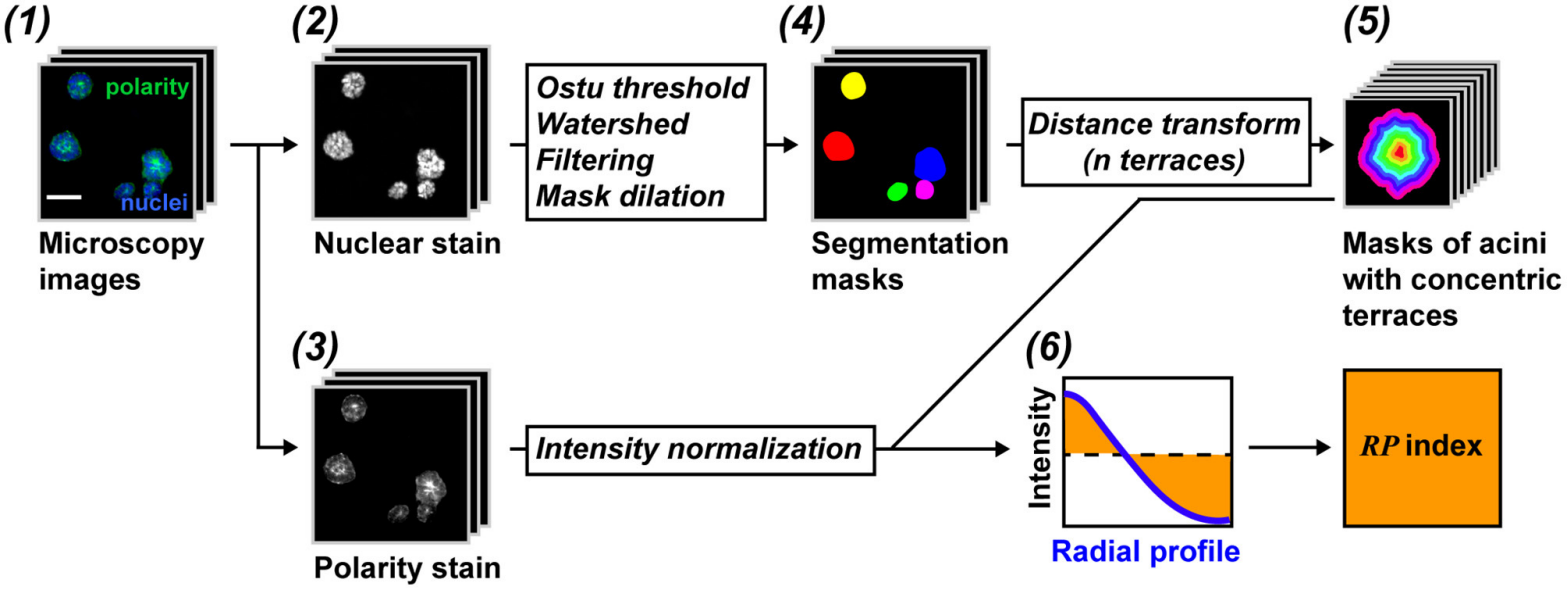
RadialProfiler flowchart. (1) Images are taken from acini cultures stained with a nuclear dye (2) and for a cell polarity marker (3). (4) Acini are segmented based on the DNA dye. Filtering steps exclude structures with inappropriate sizes or structures that are out of focus. (5) Acini are divided into concentric terraces used to calculate radial profiles of polarity. (6) The profiles are normalized and integrated to obtain a summary value of polarity (*RP* index). Scale bar, 50 μm. See text for details.

RadialProfiler identifies and segments single or grouped acini based on a nuclear stain and separates contiguous acini with a watershed algorithm. A filtering step excludes structures smaller or larger than set values, as well as blurred, out-of-focus, acini. Regions of interest (ROIs) corresponding to individual acini are divided into concentric terraces. The number of terraces depends on the size of the acini and the magnification used to capture images. It is set by the user. The concentric terraces are then used to calculate a radial profile of polarity for each acinus. The intensity profiles are normalized to avoid influences from the staining procedure. In addition, the center of the acinus is defined with a radial value of zero and the periphery as a radial value of one, thereby avoiding effects linked to acini sizes. A flowchart of the analysis is shown in Figure 2. Steep radial profiles represent polarized structures, whereas more horizontal curves represent non-polarized acini. Radial polarity indexes (*RP*) are calculated from the RP curves for direct comparisons between treatment conditions according to the equation:

(1) $$\begin{array}{r} RP=\overset{n}{\underset{i=1}{\sum }}|1-RP_{i}| \end{array}$$

Here, *RP*_*i*_ is the radial polarity of the *i*th terrace. The higher the value of the *RP* index, the more centrally concentrated is the polarity marker. Lower *RP* values indicate the polarity markers are more evenly distributed radially. To distinguish between apical and basal marker distributions, positive or negative signs are assigned to *RP* indexes. By definition, *RP* indexes from descending curves (apical) are set to positive values, whereas upward RP curves (basal) yield *RP* indexes with negative values. RadialProfiler was initially implemented in ImageJ (http://rsbweb.nih.gov/ij/) [see (35)], using an approach inspired by the Radial Profile Plot plugin from Paul Baggethun (https://imagej.nih.gov/ij/plugins/radial-profile.html). The algorithm was then translated for MATLAB and the following key improvements were made: (1) addition of watershed to improve threshold-based segmentation, (2) dilation of the identified acini to account for the discrepancy between borders of nuclear-stained images as opposed to true membrane edges, (3) substitution of approximated circles with contour terracing to calculate radial profiles, and (4) addition of an exclusion criteria based on image blur to exclude out-of-focus acini. The RadialProfiler workflow is summarized below.

### Image Segmentation

Nuclear stain images are smoothened (by replacing each original pixel intensity value with the average intensity value corresponding to a 3 × 3 kernel size). This step reduces noise before initial segmentation, which is based on the global Otsu thresholding method. Initial segmentation usually leaves errors, such as under-segmentation, where two or more adjacent acini are joined into one, larger ROI in the binary mask. To separate merged structures, the algorithm applies a watershed on the binary mask obtained from Otsu thresholding. Before watershedding is applied, the borders of the identified ROIs are smoothened. To create an image for a watershed, a distance function is performed on the binary mask that reports the distance of each interior pixel to the nearest border pixel, and regional minima are found. The MATLAB watershed function is applied on this distance image, and pixels labeled as 0 in the resulting matrix are then labeled as 0 in the binary image. Finally, acini ROIs are dilated by a certain number of pixels depending on the image magnification. This is done as the true membrane edge of the acinus lies outside of the ROI identified based on the nuclear stain.

### Filtering

**Figure 3:**
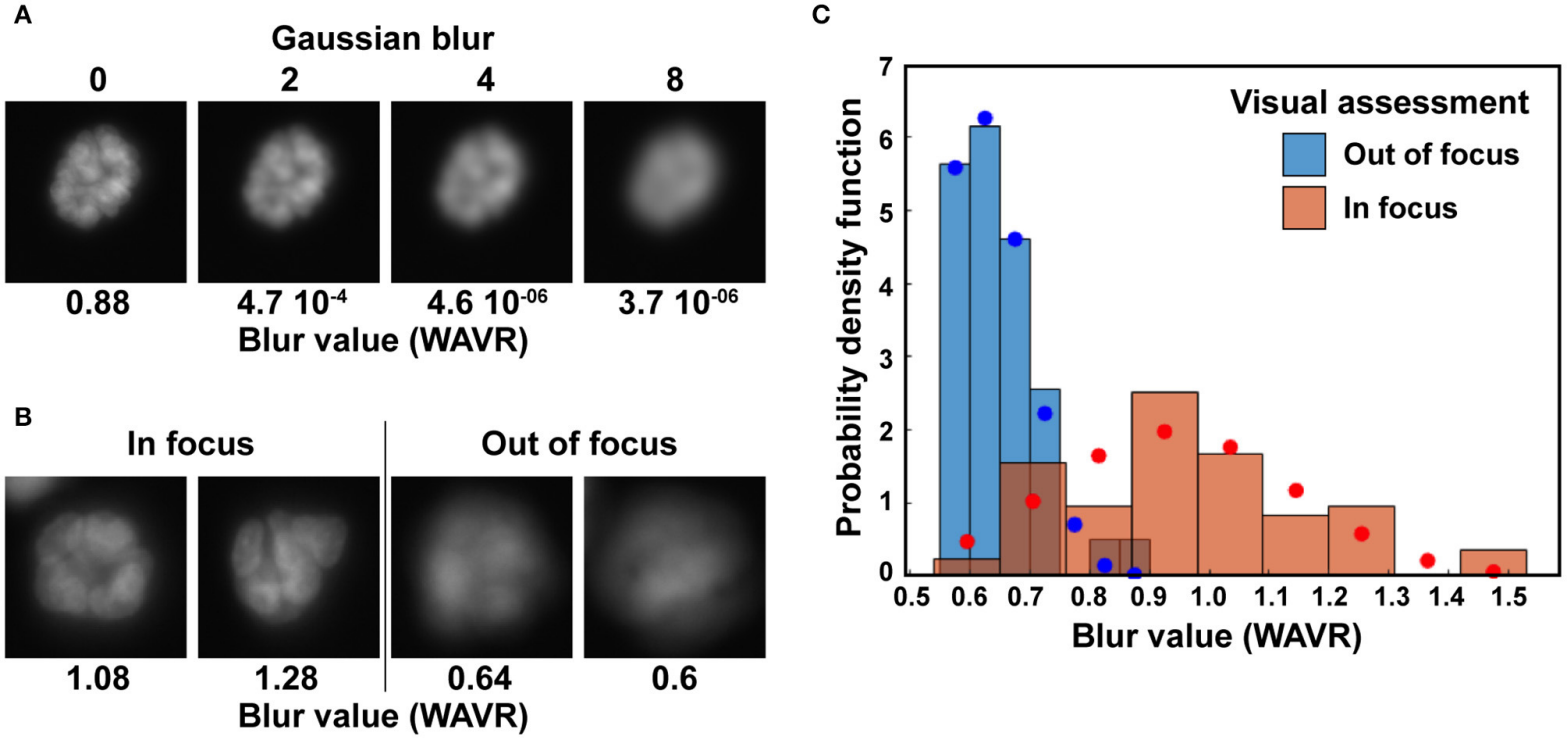
Elimination of out-of-focus acini. **(A)** Illustration of the wavelets (WAVR) blur metric calculated for Hoechst images with different levels of Gaussian blur. **(B)** Representative acini images (Hoechst stain) deemed either in focus or out of focus and their corresponding WAVR values. Images in A and B were taken with an epifluorescence microscope at 20× magnification, using a sCMOS camera. **(C)** Histograms showing the probability density function of WAVR values belonging to acini images visually rated as clear (in focus; *n* = 76) or blurry (out of focus; *n* = 39). Corresponding Levenberg-Marquardt fits of normal distributions are shown in blue circles and red circles for the two distributions.

Binary masks are filtered to exclude (1) structures partially on the border of an image, (2) structures with sizes outside a specified range, and (3) structures for which the level of blur is above a user-defined cutoff. Multiple algorithms have been developed to quantify blur in an image. We compared the different approaches summarized by Pertuz et al. (43) to determine which algorithm performed best at distinguishing blurred, out-of-focus acini based on nuclear stain images. Different levels of Gaussian blur were applied to a subset of images, creating series of images with defined levels of blurriness (Figure 3A). We also visually assigned acini from wide field microscopy images to clear and blurry categories (Figure 3B). For both approaches, we found that a wavelet-based operator (WAVR in the Focus Measure MATLAB function) was highly sensitive to Gaussian blurring and performed best to parse in-focus from out-of-focus acini. A plot summarizing the results is given Figure 3C. The graph shows the WAVR probability density function for acini visually characterized as either in focus or out of focus, revealing low WAVR values for blurry structures. The WAVR values determined from a Gaussian fit were 0.61 ± 0.08 and 0.94 ± 0.2 (mean/SD; *P* < 0.00001, Student's *t*-test) for out-of-focus and in-focus images, respectively. In this example, using a WAVR cutoff of 0.8 lead to the correct identification of 95% of acini deemed out of focus by visual evaluation, while retaining 78% of the structures visually assigned as in focus. This demonstrates that the WAVR blur value effectively distinguishes in-focus from blurry images.

### Contour Terracing

Our previous algorithm (35) discarded all acini that were not highly circular in shape because concentric circles were used to assign image pixels to the different radial zones. To lessen the amount of excluded acini and to improve precision, the current RadialProfiler algorithm defines concentric “terraces” within each acinus. This step is performed using a distance transformation similar to the one used for the watershed technique. The distance transformation uses the binary mask (ROI) of an acinus. For each true pixel, the transformation returns the Euclidean distance between that pixel and the closest edge of the structure (i.e., the ROI boundary). By analogy, each acinus is treated as a “mountain,” where the edges have lowest height, and the center marks the highest elevation. Acini ROIs are converted into topographical maps with contour lines (or terraces) of equal height ranges going from the base to the peak. Having a set number of terraces (radial bin values in the software interface) is important to normalize results for comparisons between different acini of unequal sizes and between treatment conditions.

### RP Index Calculation

To calculate RP curves, the terraces defined in the previous step are imposed on the polarity images. The average pixel intensity in each terrace is calculated and divided by the average pixel intensity for the entire acinus. This normalization step yields RP curves that are not dependent on the staining efficacy (which can be uneven). The number of points for these curves is equal to the number of terraces selected. To obtain an *RP* index value for each acinus, each of the normalized radial intensities (*RP*_*i*_) are subtracted from one (the average) and the corresponding absolute values are summed—see Equation (1). A negative sign is added to *RP* indexes from RP profiles with a positive slope, to distinguish between apical and basal signal localization.

## Analyses of epithelial polarity using RadialProfiler

The RadialProfiler algorithm was developed to analyze acini produced with non-neoplastic HMT-3522 S1 breast epithelial cells (44). We expect that the radial profile method is applicable to acini produced with other normal or pre-malignant epithelial cell lines. Detailed protocols for 3D cell culture of breast acini can be found in ref. (38). Briefly, a thin coat of rBM (e.g., Corning Matrigel™) is applied at the bottom of the culture vessel. Then, a single cell suspension (42,000 cells/cm^2^) is added on top of the rBM coat and is overlaid with rBM diluted in culture medium (5% final concentration) to engage the cell surface integrins that are not in contact with the rBM-coated substratum, and to promote the development of 3D structures. Different culture vessels (35 mm dishes, chambered slides, multiwell plates) are used depending on the analysis method (fixed vs. live imaging), and the throughput level (low vs. medium). For live imaging in glass-bottom dishes and plates, a thinner coat of rBM is applied to enable imaging with high numerical aperture (NA) objectives, which typically have relatively short working distances (<0.2 mm).

RadialProfiler can be applied to quantify epithelial markers detected by immunofluorescence [as described in (35)], or to quantify cortical actin labeled in live acini with the SiR-actin dye (Cytoskeleton Inc.). DAPI and Hoechst are used to counterstain cell nuclei in fixed and live experiments, respectively.

For imaging, our laboratory uses an automated IX83 microscope (Olympus) equipped with a motorized ultrasonic stage and a TruFocus Z drift compensation module. For RadialProfiler analyses, images are taken with either 10× (NA = 0.3) or 20× (NA = 0.45) air lenses, using a sCMOS camera (ORCA-Flash4.0, Hamamatsu). The RadialProfiler software was also tested with images acquired using different imaging systems, including a high content imager (Perkin Elmer Operetta CLS). RadialProfiler and the underlying approach to analyze polarity are agnostic to the imaging system. Fields of view are chosen either in an automated fashion or based on nuclear signals (DAPI or Hoechst) to avoid bias. For live cell analyses, acini are maintained at 37°C and 5% CO_2_ using a stage-top incubator (Tokai Hit). The minimal resolution needed depends on the number of radial terraces used by RadialProfiler. To improve statistical power, the number of acini in a single image needs to be maximized, which can be achieved with a low magnification objective. However, the ability to analyze the distribution of polarity markers in an acinus improves with the number of sampled image points. Lenses with higher magnification generally provide higher resolution images, with more pixels per acini, albeit with fewer acini in each field of view. In the end, the choice of magnification is directed by the need to have an individual acinus sampled at enough camera pixels to allow an accurate polarity radial profile analysis with a suitable number of terraces. We determined that using 5–10 bins that are two pixels wide yields accurate measurements. This corresponds to a diameter of 20–40 pixels, which, for a circular acinus, corresponds to 316–1,264 pixels. Acini are not perfect spheres; this value is therefore an estimate. This has been reinforced empirically through our analysis of large data sets.

**Figure 4:**
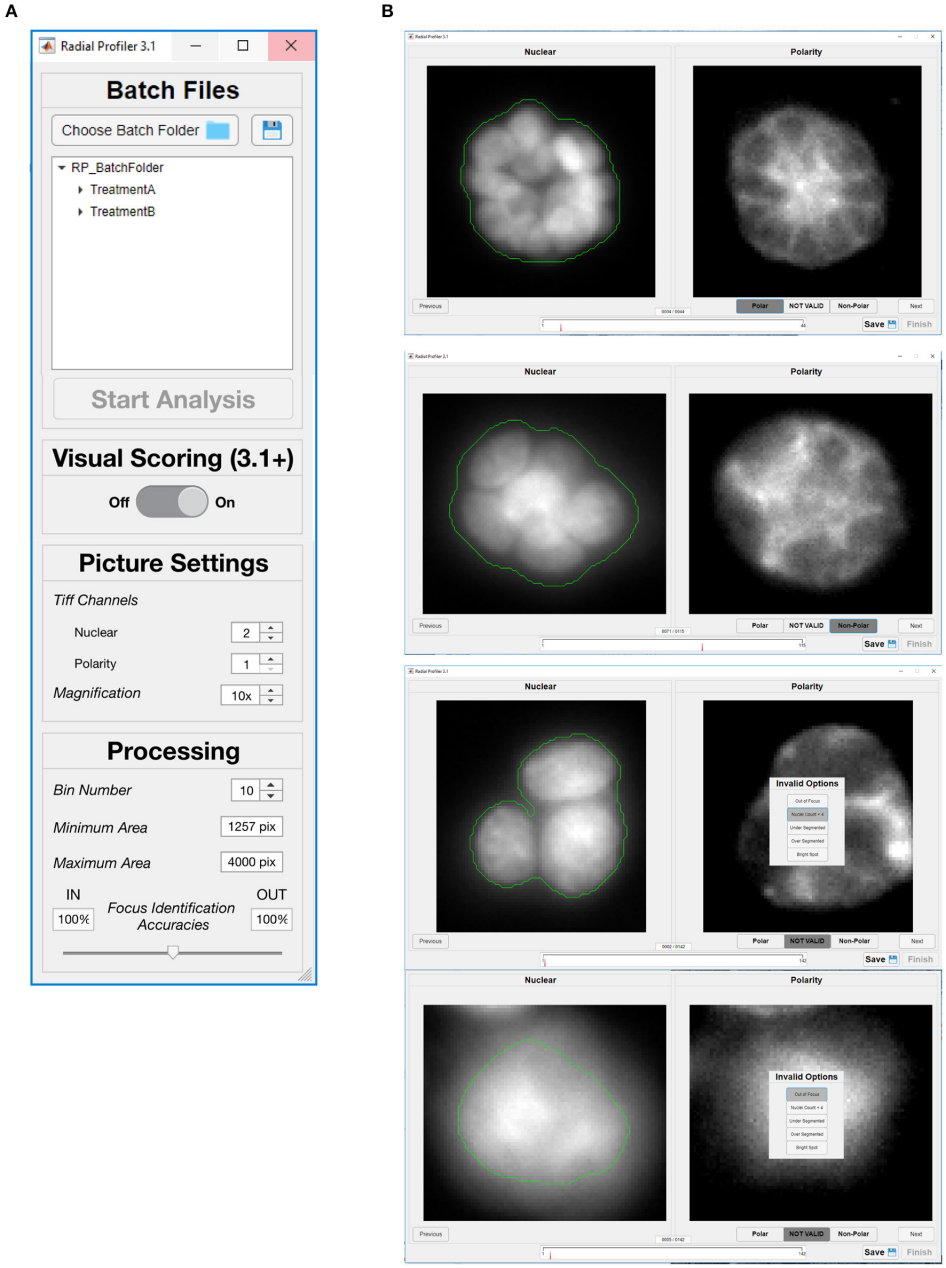
Graphical user interfaces of RadialProfiler. **(A)** Window to select image folders corresponding to the dataset for analysis, and to define analysis parameters. The user chooses between supervised and unsupervised analyses with this first dialog box by turning visual scoring on or off. **(B)** Interface assisting visual scoring of polarity marker distribution. This window appears when the user selects supervised analysis. For each acinus identified by RadialProfiler (in the entire dataset selected in **A**), nuclear stain and polarity images are displayed side-by-side. The user input is a binary choice between (“Polar” or “Non-Polar”) or exclusion from analysis. The progress bar (bottom) indicates the number of structures that remain to be scored. Acini appear in a randomized order. See text for details.

**Figure 5:**
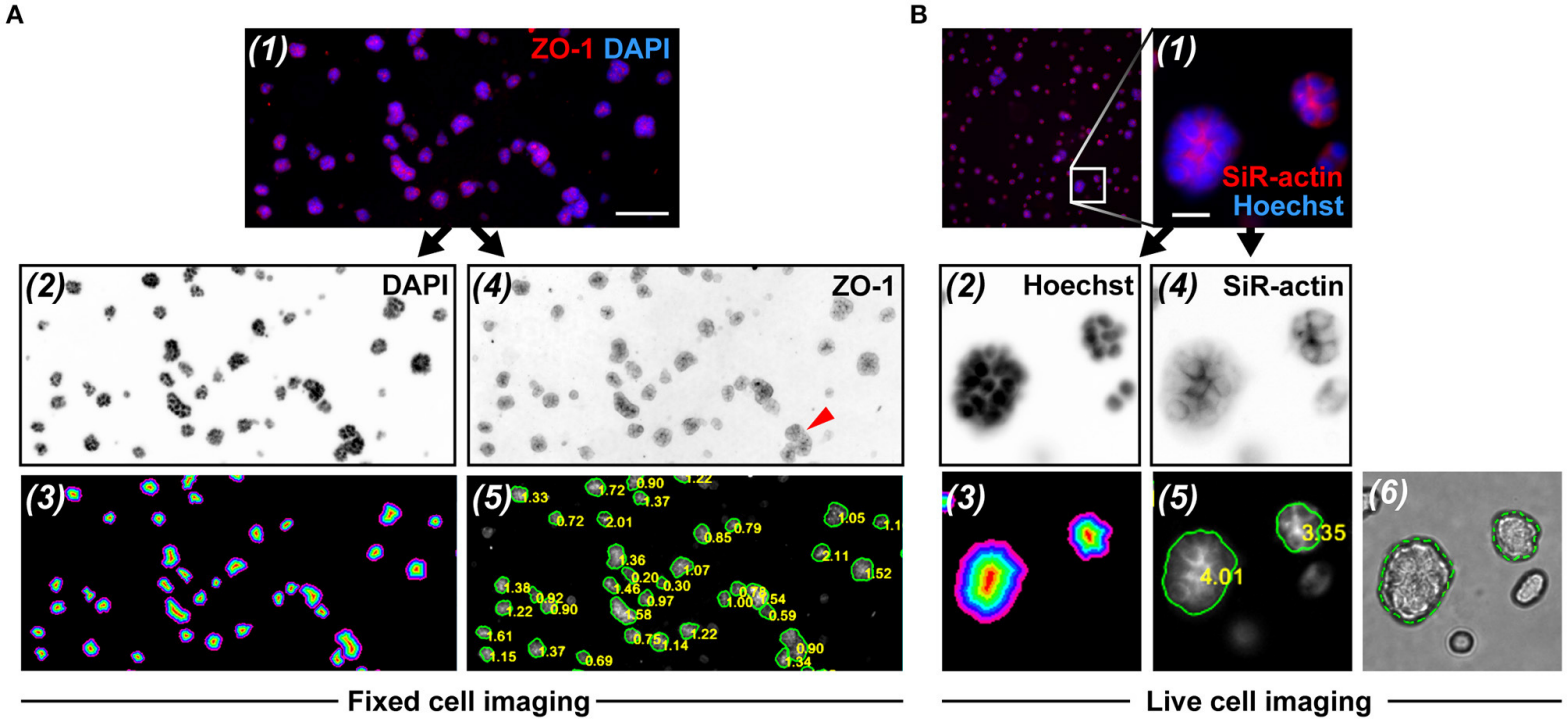
RadialProfiler analysis of wide field fluorescence images from fixed and immunostained acini **(A)**, or of cortical actin staining in live acini **(B)**. The figure shows (1) portions of overlay images, (2) nuclear stain images (inverted to improve visualization), (3) corresponding masks with the concentric terraces, (4) inverted polarity images, and (5) polarity images annotated with acini contours and *RP* indexes. In rare instances (red arrowhead in A-4), acini were under-segmented. In B, an overlay of the bright field image and the corresponding contour ROI validates the segmentation (6). Scale bars, 100 μm **(A)** and 20 μm **(B)**.

**Figure 6:**
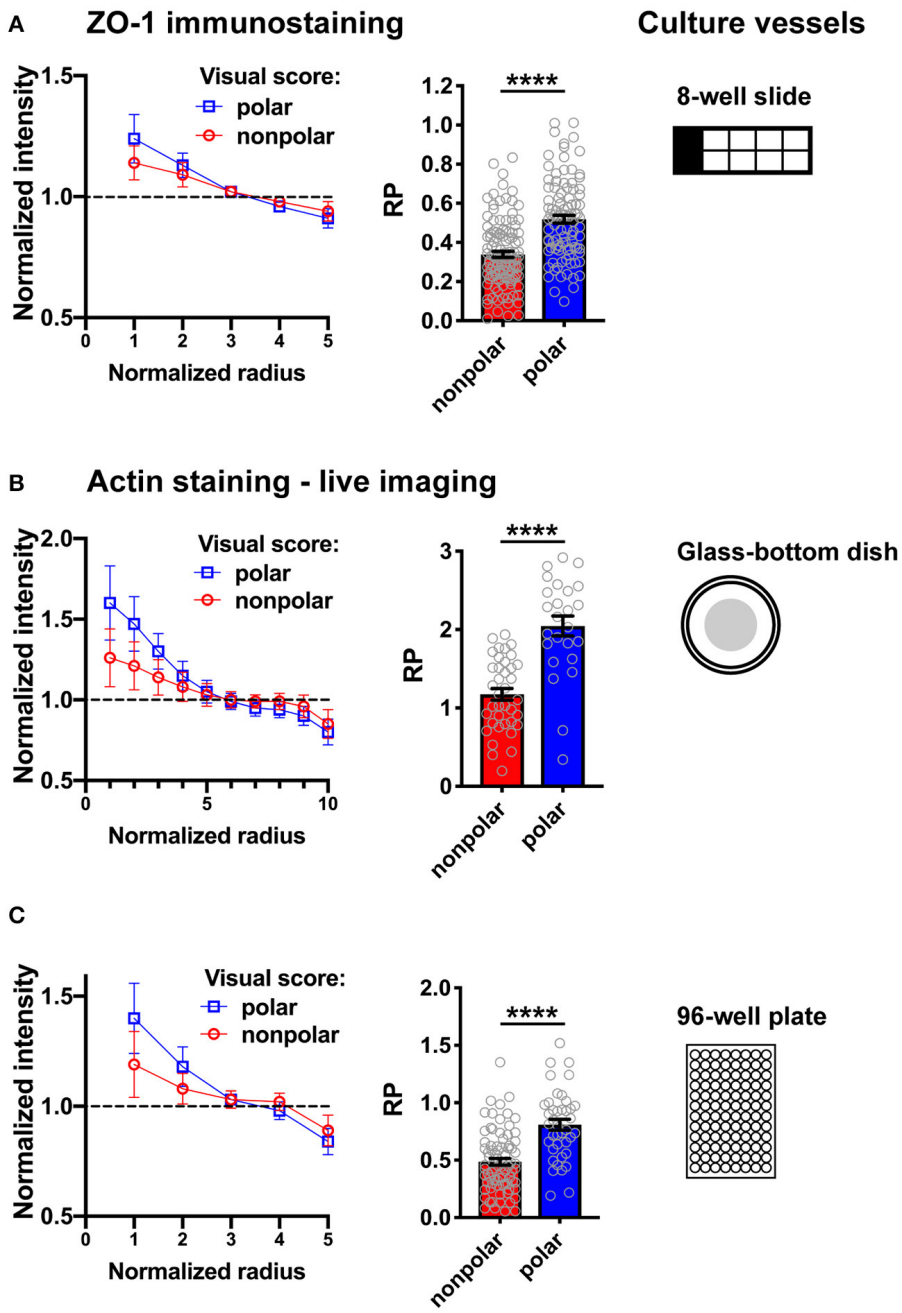
Illustration of RadialProfiler results for HMT-3522 S1 acini in different culture vessels. The supervised version of the software was used to classify acini in polarized and non-polarized categories. Radial profiles (left) and bar graphs of the *RP* indexes (right) are shown for both categories. **(A)** Fixed acini immunostained for ZO-1. **(B,C)** Live imaging of acini stained with SiR-actin. Fluorescence images were captured using a wide field microscope (Olympus; **A,B**) or with an automated spinning disc high content imaging system (Perkin Elmer Operetta; **C**). In C, maximal intensity projections of 10 confocal frames were analyzed. The number of radial bins used for analysis was adapted to the different magnifications and image resolutions. ****P < 0.0001 (Student's *t*-test).

RadialProfiler operates in two modes, either supervised or unsupervised. The user chooses between these two modes with the first dialog box (Figure 4A). The unsupervised mode runs the analysis automatically once the program parameters are set. It retrieves a table listing the normalized radial intensity values and an *RP* index value for each acinus. It also produces images annotated with segmentation results and *RP* index values (Figure 5). Results in the table are grouped by experimental conditions. The supervised version performs the same calculations as the unsupervised version but also includes a graphical user interface (Figure 4B), allowing the investigator to visually score polarity and assess the quality of the acini identification steps (segmentation efficacy, blurriness, etc.). Individual acini are presented to the user in a randomized order and without providing any treatment information, which enables blind scoring. After completion of visual scoring, a table with *RP* index values and user scores is produced. Additional details on RadialProfiler installation and usage are provided as Supplementary Information to this article. Representative results are shown in Figure 6.

## Discussion

We developed a method to quantify epithelial polarity in breast acini organoid cultures. The method is based on radial marker profiling and results in a single polarity index to assess establishment or breakdown of apical-basal polarity in populations of acini. This method should be applicable to a wide variety of cell types and treatment conditions. The software interface is user-friendly and circumvents the need to use command lines in MATLAB. RadialProfiler is therefore accessible to biologists and health scientists with minimal knowledge of the computing platform. Importantly, the *RP* index produced by the software successfully distinguishes between non-polar and polar acini, as demonstrated in the analyses presented in Figure 6. Similar results were obtained using different imaging platforms.

S1 cell acini are characterized by a small lumen; hence, radial profile curves of apically polarized structures have a maximal value close to the center of the structure. For epithelial cells forming cysts with a larger lumen (e.g., MDCK cells), radial profile maxima will be shifted toward the periphery. In this case, disruption of polarity protein distribution will still alter radial profiles, although we do not expect the method to perform well for structures with a very large lumen. Whereas the *RP* index distinguishes well between apical and basal signals (high positive vs. high negative values), as well as between polarized and uniform signals (high vs. low values), the index is not very sensitive to smaller radial shifts and may not be appropriate to quantify markers with multimodal distributions. The shape of the radial profile curves is however more indicative, and additional curve characteristics can be considered, such as the number of intersections with the average line (*x* = 1), the radial position of the maxima, etc.

RadialProfiler was developed and validated for homotypic cultures of breast acini. Co-cultures including multiple cell types (fibroblasts, adipocytes, immune cells) are better models—albeit more complex—to capture the effects of epithelial-stromal cell interactions on drug pharmacokinetics and phenotypical outcomes (35, 45–47). Acini in co-culture systems can in principle be analyzed using RadialProfiler, as long as epithelial cells can be distinguished from other cell types. For example, a breast epithelial cell line stably expressing a GFP-tagged histone can be co-cultured with other cell types (untagged or tagged with a different chromophore). In this case, GFP signals would be used instead of Hoechst staining to identify and segment acini with the current version of the RadialProfiler. The presence of other cell types should not interfere with immunostaining or cortical actin staining in the acini. Staining of the breast epithelial cells prior to co-culture with a cell tracking dyes would be an alternative approach.

We welcome feedback on RadialProfiler performance in different contexts and plan on further developments for this approach. In particular, operation of RadialProfiler in supervised mode yields rich datasets annotated for polarity by expert investigators. Datasets from supervised analyses also contain information on segmentation and image blur. This “ground truth” information will enable us to integrate machine learning into the next version of the algorithm. RadialProfiler is currently limited to the analysis of acini cultures *in vitro*. However, the general principle to quantify radial profiles is applicable to tissues, and we plan on further developing the computational approach for tissue analyses.

We hope and anticipate that this assay will fill unmet needs in primary prevention of breast cancer and other carcinomas, with applications including (1) chemoprevention drug screening, (2) toxicology assessment of suspected carcinogens and pharmacological lead compounds, and (3) personalized cancer risk diagnosis. High content screening methods for cancer prevention are scarce. Since loss of apical-basal polarity is an early step enabling the initiation of carcinoma, an assay of epithelial polarity may be used to screen for chemoprevention drugs or natural compounds preventing polarity loss or restoring polarity. The RP assay may also be implemented to weed out drug candidates with toxic effects on the epithelial architecture before testing in mice models. Indeed, the vast majority of hit compounds in drug discovery pipelines fail the transition from the initial screen to animal models. Relevant *in vitro* assays, such as the RP assessment, may be used to rapidly and cheaply screen for toxic effects on normal cells, thereby reducing the need for animal research, which is expensive and raises ethical concerns. More broadly, assays with non-neoplastic cell organoids can be used to assess suspected carcinogens (48–51).

Current breast cancer risk assessment methods provide population-based estimates of risk rather than personalized risk assessment. Genetic testing can identify mutations associated with cancer risk (e.g., BRCA1/2 for breast cancer), yet only a small fraction of malignancies (about 5% for breast cancer) have a known genetic origin. Cell-phenotypical assays, including epithelial polarity readouts, may be used to rapidly assess personalized breast cancer risk, for example for women participating in lifestyle interventions. In these cases, acini cultures and RP analyses may serve as biomarkers for integrative assessment of improvements in metabolic risk factors.

## Data Availability Statement

The RadialProfiler MATLAB code, as well as a detailed tutorial describing installation and use of RadialProfiler and test images of breast acini is available on OSF (https://osf.io/g48ac/). The latest version of the code can also be downloaded from the MathWorks website.

## Author Contributions

LM wrote the computer code. JH performed the experiments. KB and P-AV directed the research and wrote the manuscript. LM and JH edited the manuscript.

### Conflict of Interest

KB and P-AV have filed a patent application on the use of the radial profile method to measure epithelial polarity. The remaining authors declare that the research was conducted in the absence of any commercial or financial relationships that could be construed as a potential conflict of interest.
