# Supplementary material for: Radial Profile Analysis of Epithelial Polarity in Breast Acini: A Tool for Primary (Breast) Cancer Prevention
Source: Front Med (Lausanne). 2020 Jan 10;6:314. doi: 10.3389/fmed.2019.00314 (PMC6970192; doi:10.3389/fmed.2019.00314)
Supplement: Supplementary file 1 [file Data_Sheet_1.pdf]

SUPPLEMENTAL INFORMATION

Radial profile analysis of epithelial polarity in breast acini cultures: a tool for primary (breast) cancer prevention

Lawton Manning[1], Julia Holmes[2], Keith Bonin[1,3], and Pierre-Alexandre Vidi[2,3]

[1] Department of Physics, Wake Forest University, Winston-Salem, NC 27109, USA.
[2] Department of Cancer Biology, Wake Forest School of Medicine, Winston-Salem, NC 27157, USA.
[3] Comprehensive Cancer Center of Wake Forest University

Instructions to install and use RadialProfiler

Instructions for the latest version of RadialProfiler can be found with the code at OSF (https://osf.io/g48ac/).

1. Install the MATLAB code for RadialProfiler on the hard drive of the computer. RadialProviler was written for MATLAB R2018b.
2. Images for analysis need to be TIFF stacks including a DNA stain and polarity marker. The order can be specified in the RadialProfiler graphical user interface (GUI) and additional layers may be included in the TIFF stack (that will be ignored). Organize images for analysis according to the following folder structure:

```
MATLAB_RP
        |__ RP_BatchFolder
              |__ ConditionA
                    |__ ConditionA-image1.tif
                    |__ ConditionA-image2.tif
                    |__ etc.

              |__ ConditionB
                    |__ ConditionB-image1.tif
                    |__ ConditionB-image2.tif
                    |__ etc.
```

ConditionA, B, etc. are different experimental treatments that need to be analyzed separately.

RadialProfiler can also handle the following folder structure:

```
MATLAB_RP
    |__ RP_BatchFolder
            |__ Experiment1
                    |__ ConditionA
                            |__ ConditionA-image1.tif
                            |__ ConditionA-image2.tif
                            |__ etc.

                    |__ ConditionB
                            |__ ConditionB-image1.tif
                            |__ ConditionB-image2.tif
                            |__ etc.

                    |__ etc.

            |__ Experiment2
                    etc.
```

The results are saved in the parent folder (MATLAB_RP).

3. To begin analysis, add both RP_universal and MATLAB_RP folders and subfolders to the MATLAB path.
4. Locate the **‘start’** script in the folder containing the RadialProfiler code and double-click on it to launch the GUI.
5. Once the GUI has loaded, use ‘**Choose Batch Folder**’ to select the parent folder (RP_BatchFolder in the file arborescence illustrated above) that contains the image folders for each condition (at least one).
6. The **‘Visual Scoring’** switch allows for toggling between supervised (ON) or unsupervised (OFF) analyses. Leaving this switch on will prompt user evaluation of software-identified structures. Turning the switch off will direct the software to perform a fully automated analysis of epithelial polarity marker distribution, without any visual input from the user.
7. Under **‘Picture Settings’**, select which layer (or channel) of the TIFF stack corresponds to the nuclear stain (e.g. DAPI, Hoechst) and which layer corresponds to the polarity marker image. Also select the magnification that was used for imaging.
8. Under **‘Processing’**, select the number of **‘Bins’** (or radial ‘shells’). Note that bins need to be at least two pixels thick. The default bin number of 10 will separate an identified acinus into ten equidistant, concentric regions. The optimal bin number needs to be determined empirically. Choosing a high bin number with low magnification images (few pixels for each acinar structure) will result in most acini being discarded.

9. Under **‘Processing’**, set minimal and maximal sizes (in pixels) for acini detection. Default values (minimum = 1257 pix, maximum = 4000 pix) are optimized for detection of breast acini imaged at 10X magnification.
10. Under **‘Processing’,** The ‘Focus Identification Accuracies’ slider allows the user to modulate how stringently the software discards blurred acini. Sliding this indicator to the extreme right will direct the software to discard acini with any detectable blur, while sliding the indicator to the extreme left tells the software to be more lenient when assessing blur, including more structures overall.
11. Click **‘Start Analysis’** and wait until MATLAB finishes segmentation and randomization of acini in all images. This may take a few minutes depending on the number of images. Note that image series containing a large number of identifiable acini (greater than 1000) can take ten minutes or more to load. If ‘Visual Scoring’ was turned off, the software skips human analysis, straight to the generation of results in a CSV file. If ‘Visual Scoring’ was on, each acinus identified in the images gets displayed sequentially (and in a randomized order) in a new window, with nuclear stain (left) and polarity marker (right) side-by-side. Investigators score visually the distribution of polarity markers (‘Polar’ vs. ‘Non-Polar’), or choose ‘NOT VALID’ and select on the appropriate subcategory (‘Out of Focus’, ‘Nuclei Count < 4’, ‘Under Segmented’, ‘Over Segmented’, ‘Bright Spot’).
12. The bar at the bottom indicates the total number of acini and scoring progress.
13. Arrow keys can be used instead of mouse clicks:

    Up (or ‘w’) = Polar

    Down (or ‘s’) = Non-Polar

    Left (or ‘a’) = Previous (to go back to acini already scored)

    Right (or ‘d’) = Next

    x = NOT VALID (then use the mouse to select between options)

14. To stop and resume visual scoring later, click **‘Save’**. When resuming analysis, start the GUI as above and press the save button icon (next to ‘Choose Batch Folder’). This will convert the ‘Start Analysis’ button to ‘Continue Analysis’. When choosing the batch folder again, select the one that has “-IN-PROGRESS” appended to its name. This folder contains all of the individually segmented acini as separate TIFFs that still need to be analyzed. Next, press ‘Continue Analysis’ and the software will resume on the last unscored acinus in the same random order.
15. Once the last acinus has been scored, click **‘Finish’**.
16. In the parent folder (MATLAB_RP), a comma-separated values (CSV) file is saved that contains both RP and visual scoring values. A new folder is also created with individually cropped acini. The metadata of these TIFF files contain visual assessment values (if processed in supervised mode).
